# Multicenter study of levodopa carbidopa intestinal gel in Parkinson’s disease: the Turkish experience

**DOI:** 10.3906/sag-1904-150

**Published:** 2020-02-13

**Authors:** Murat GÜLTEKİN, Çağrı ULUKAN, Sabiha TEZCAN, Okan DOĞU, Haşmet HANAĞASI, Başar BİLGİÇ, Ayşe BORA TOKÇAER, Raif ÇAKMUR, Bülent ELİBOL, Meral MİRZA, Dilek İNCE GÜNAL, Çiğdem Sevda ERER ÖZBEK, Gülay KENANGİL, Özge YILMAZ KÜSBECİ, Muhittin Cenk AKBOSTANCI

**Affiliations:** 1 Department of Neurology, Faculty of Medicine, Erciyes University, Kayseri Turkey; 2 Department of Neurology, Faculty of Medicine, Ankara University, Ankara Turkey; 3 Department of Neurology, Faculty of Medicine, Mersin University, Mersin Turkey; 4 Department of Neurology, Faculty of Medicine, İstanbul University, İstanbul Turkey; 5 Department of Neurology, Faculty of Medicine, Gazi University, Ankara Turkey; 6 Department of Neurology, Faculty of Medicine, Dokuz Eylül University, İzmir Turkey; 7 Department of Neurology, Faculty of Medicine, Hacettepe University, Ankara Turkey; 8 Department of Neurology, Faculty of Medicine, Marmara University, İstanbul Turkey; 9 Department of Neurology, Faculty of Medicine, Uludağ University, Bursa Turkey; 10 Department of Neurology, Medical Park Göztepe, Bahçeşehir University, İstanbul Turkey; 11 Department of Neurology, Bozyaka Training and Research Hospital, İzmir Turkey

**Keywords:** Parkinson’s disease, levodopa carbidopa intestinal gel, efficacy

## Abstract

**Background/aim:**

Our purpose was to determine the efficacy of levodopa carbidopa intestinal gel (LCIG) in a series of Turkish patients with Parkinson’s disease (PD).

**Materials and methods:**

We had telephone calls with 54 patients from 11 neurology centers who were on LCIG treatment, and 44 patients or their caregivers were included in an eight-item survey between September 2015 and June 2016. The reliability and validity of the survey were evaluated with intraclass correlation coefficients for every question separately.

**Results:**

Average age of the patients were 63.48 and the duration of PD was 12.79 years. Average LCIG treatment period was 15.63 months. Percentages of the patients who reported they were ‘better’ after LCIG treatment were as follows: 80% for time spent off, 55% for dyskinesia, 65% for tremor, 85% for gait disorder, 50% for pain, 50% for sleep disorders, 42.5% for depression, 32.5% for incontinence, and 70% for activities of daily living. Cronbach’s alpha was 0.795 and the intraclass correlation coefficient was reliable for the items.

**Conclusion:**

As detected by a survey performed by telephone calls with good interrater reliability, patients with PD improve with LCIG treatment in many aspects of the disease.

## 1. Introduction

Parkinson’s disease (PD) is the second most common progressive neurodegenerative disease occurring as a result of the dopaminergic neuron loss in the nigrostriatal pathway [1]. There can be motor as well as nonmotor symptoms in PD. As the disease progresses, the patients begin to need device-aided treatments such as apomorphine pump, deep brain stimulation (DBS), and levodopa/carbidopa intestinal gel (LCIG). Thanks to these treatments, both motor and nonmotor symptoms of the patients are significantly treated and activities of daily living (AoDL) become more comfortable [2].

Dopamine replacement with its oral precursor levodopa (L-DOPA) to stimulate striatal dopamine receptors is the gold-standard treatment of PD [3]. However, the therapeutic window of levodopa narrows progressively within years and most patients develop motor complications eventually, such as motor fluctuations and dyskinesia [4]. Due to its short plasma half-life, oral levodopa treatment cannot stimulate receptors in a continuous manner and causes oscillations of plasma levels, which are closely correlated with motor fluctuations [5]. In this period of PD, patients may need device-aided treatments. LCIG is a suspension administered to the jejunum directly via percutaneous gastrojejunostomy tube to achieve more stabilized plasma levodopa levels. Based on multiple studies LCIG is effective in eliminating motor and nonmotor fluctuations and improving quality of life in advanced stages of PD [6–8]. 

In this study, we aimed to evaluate the efficacy of LCIG treatment for PD on motor and nonmotor symptoms and AoDL via a structured survey as a multicenter study.

## 2. Materials and methods 

The Turkish Duodopa study was a cross-sectional, descriptive study designed to evaluate the efficacy of continuous LCIG infusion via a previously prepared survey to compare former and subsequent treatment of motor and nonmotor symptoms. This study was conducted between September 2015 and September 2016. Eleven neurology centers across Turkey were included in the study. The survey comprises a total of 9 questions. Four questions are related to motor symptoms (time spent off, dyskinesia, tremor, gait disorder), 4 questions are related to nonmotor symptoms (pain, sleep disorders, depression, incontinence), and one is related to AoDL. Questions in the survey are given in Appendix 1. Patients’ demographics, PD onset, and LCIG treatment duration were collected prospectively. 

Fifty-four patients who were receiving LCIG were evaluated for this study. We could not enroll 10 patients because of missing contact information (n = 6), unwillingness to participate in the survey (n = 1), termination of therapy at the time of contact for loss of appetite (n = 2), and inadequate treatment response (n = 1). A total of 44 patients were evaluated. The patients and their caregivers were then asked to answer the survey on the telephone. Patients and their caregivers were asked to scale the effect of LCIG on every symptom, comparing the periods before and after the treatment as ‘better’, ‘same’, ‘worse’, ‘don’t know’, or ‘did not have this symptom’. Results were expressed as absolute numbers and frequencies. All interviews were recorded and two neurology residents listened to and evaluated these interviews separately. The protocol of the study was approved by the Ethics Committee of Ankara University.

### 2.1. Statistics

To compare the variables before and after LCIG initiation, the Wilcoxon matched pairs signed-rank test was used. The survey’s reliability and validity were assessed with Cronbach’s alpha and intraclass correlation coefficients (ICCs; interrater reliability) (Table 1). The statistical significance level was 0.05. SPSS 23.0 for Mac (IBM Corp., Armonk, NY, USA) was employed for data processing and analysis.

**Table 1:** Intraclass correlation coefficient (ICC).

Item	ICC
Motor Time spent ‘off’	0.981
Dyskinesia	0.946
Tremor	0.995
Gait disorder	0.792
Nonmotor pain	0.980
Sleep disorders	1.000
Depression	0.958
Incontinence	0.991
Activities of daily living	0.995

## 3. Results

We collected the patient data from 11 centers across Turkey specialized on movement disorders, and we tried to reach all the patients who were receiving LCIG treatment on the telephone. Twenty-two of 44 patients (50%) were female. The mean of the age of patients, duration of PD, and LCIG treatment duration were 63.48 ± 8.4 years, 12.79 ± 9.1 years, and 15.63 ± 9.46 months, respectively. The demographic data of patients are shown in Table 2.

**Table 2:** Characteristics of the patients with Parkinson’s disease.

Parameter		Value
N		44
Age (years), mean (SD)		63.48 (8.4)
Sex, n (%)	Male	22 (50)
PD duration (years), mean (SD)		12.79 (9.1)
LCIG duration (months), mean (SD)		15.63 (9.46)

PD: Parkinson’s disease, LCIG: Levodopa carbidopa intestinal gel.

**Figure 1:**
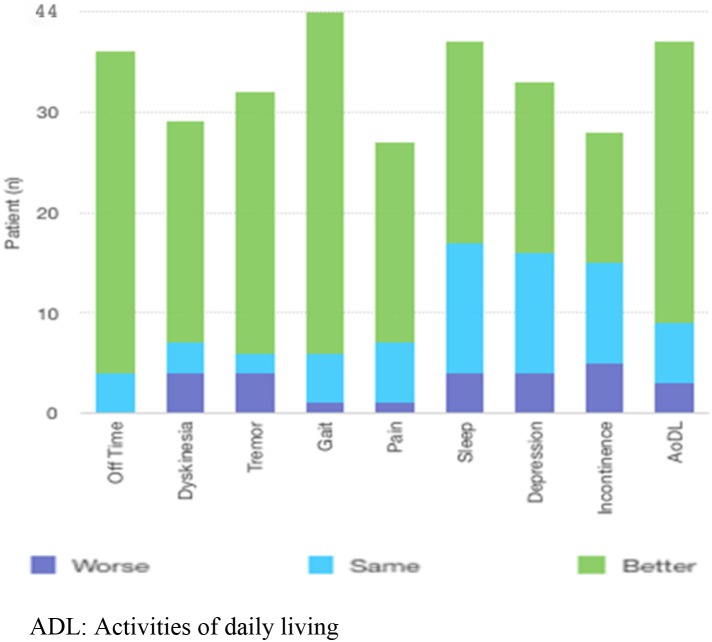
Answers given by patients to questions in the survey.

The percentages of patients who reported ‘better’ motor symptoms for time spent off, dyskinesia, tremor, and gait disorder were 80%, 55%, 65%, and 85%, respectively. The percentages of nonmotor symptoms (pain, sleeping disorders, depression, and incontinence) that improved were 50%, 50%, 42.5%, and 32.5%, respectively. The details of the answers given by the patients are presented in the Figure.

According to the patients and their caregivers’ surveys on the telephone, 70% of all patients reported that they had improved in terms of AoDL after LCIG treatment. Motor symptoms responded significantly better than nonmotor symptoms. All of the parameters investigated except incontinence were rated as significantly better. 

Cronbach’s alpha for the survey was 0.795. The reliability and validity were evaluated with the ICC for every question separately (Table 1) between raters. 

The values for Cronbach’s alpha and ICCs gave rise to the thought that the telephone questionnaire can be used as a valid and reliable tool.

## 4. Discussion 

In this study, we have conducted a quite simple survey to investigate the effect of LCIG on frequent motor and nonmotor symptoms of PD. The present study is the first study of LCIG treatment for PD patients in Turkey, with positive effects of the procedure on motor and nonmotor symptoms and AoDL.

Although there are numerous studies showing that LCIG is effective in treating PD [9–12], there is only one randomized, controlled, double-blind, double-dummy study to prove its efficacy [13]. Different types of scales (Unified PD Rating Scale: UPDRS, Parkinson Patient Quality of Life: PDQ, Nonmotor Symptoms Scale: NMSS, etc.) were employed in these studies to measure the efficacy of LCIG [14,15].

The improvement of motor symptoms was higher than nonmotor symptoms in our series. While the highest motor symptom recovery was for gait disorder (85%), highest nonmotor symptom recovery was for sleep (50%). The recovery of AoDL was found to be 50%. Some studies have also shown similar positive results [11,14]. It was demonstrated by Chang et al. that the decrease of ‘off’ time was 67% and 73% in comparison to baseline at the 6th and 12th months, respectively [6]. Fernandez et al. reported improvement of dyskinesia and at the same time increased ‘on’ time without troublesome dyskinesia at a rate of 62.9%, while the decrease of ‘on’ time with troublesome dyskinesia was 22.5% [7]. It was found by Buongiorno et al. that the efficacy of LCIG on gait disorders like falls and freezing of gait decreased the rate of such symptoms from 97% to 65% [8]. With regard to motor symptoms, the findings of the current study are compatible with the previous studies.

Băjenaru et al. evaluated 113 patients in their retrospective study conducted in nine centers located in Romania. With LCIG treatment, the ‘on’ period of the patients (period of improved mobility known as ‘on’ period) increased to 6.14 h on average, and dyskinesia periods decreased 29.4%. It was shown that the nonmotor symptoms and life quality scores of the patients improved dramatically [16]. In the study carried out by Cáceres-Redondo et al., 29 PD patients receiving LCIG treatment were monitored for two years. While similar improvements in motor and nonmotor symptoms of the patients were detected, it was revealed that although cognitive performance of the patients regressed, long-term well-being continued [17]. According to a large review published recently, the evidence about the effect of LCIG on motor fluctuations and quality of life is of average quality, and the evidence about the effect of LCIG on nonmotor symptoms is of dramatically low quality (65%–14%) [18]. The effects of LCIG on motor and nonmotor symptoms of PD are shown in Table 3 [19–21].

**Table 3:** Efficacy of levodopa carbidopa intestinal gel on motor and nonmotor symptoms.

Authors	Year	‘Off’ time	‘On’ time with dyskinesia	UPDRS II	UPDRS III	NMSST,% improvement
Honig et al. [11]	2009	-	-	11.6 ± 7.2	4.5 ± 2.2	56
Cáceres-Redondo et al. [17]	2014	32.2 mo, 24.6 ± 7.2	-	16.5 ± 5.0	29.5 ± 6.4	17
Antonini et al. [19]	2015	–4.7 ± 3.4	–1.7 ± 5.0	–3.1 ± 8.7	–3.3 ± 11.0	29
Sensi et al. [20]	2014	24 mo, 1.0 ± 0.6	-	-	24 mo, 34.7 ± 12.4	27
Bohlega et al. [21]	2015	6 mo, 1.4	-	-	19.6 ± 8.4	65

NMSST: Nonmotor Symptom Scale Total score, mo: months, UPDRS: Unified Parkinson’s Disease Rating Scale

**Table 4:** Survey form used in the study.

Evaluation of Motor and Nonmotor Symptoms	Better	Same	Worse	Unknown	She/he did not have that problem
Motor symptoms					
Is there a change when she/he is unconscious (bad)?					
Is there a change in her/his reflexes (saltatory, convulsion,spasms - not shivering) during the day?					
Is there a change in shivering?					
Is there a change her/his difficulty in walking and imbalance?					
Nonmotor symptoms					
Is there a change in aches?					
Is there a change in insomnia?					
Is there a change in senses of unhappiness, sadness, moodiness?					
Is there a change in urinary incontinence problem?					
Daily life activities					
Is there a change in her/his daily chores (eating, dressing,showering, etc.)?					
TOTAL					

There are a few limitations of our study, including a relatively small number of patients, retrieval of information through telephone calls, and a lack of information about side effects and complications. 

In conclusion, we assume that the eight items of the survey developed to assess patient response to LCIG could be used as a telephone follow-up tool, especially with patients who have difficulty reaching a hospital.

## Informed consent

All participants provided informed consent in the format required by the relevant authorities.
